# Interaction Between Modern Radiotherapy and Immunotherapy for Metastatic Prostate Cancer

**DOI:** 10.3389/fonc.2021.744679

**Published:** 2021-09-14

**Authors:** Luc Ollivier, Maureen Labbé, Delphine Fradin, Vincent Potiron, Stéphane Supiot

**Affiliations:** ^1^Institut de Cancérologie de l’Ouest, Nantes, France; ^2^Université de Nantes, CNRS, Inserm, CRCINA, Nantes, France

**Keywords:** radiotherapy, immunotherapy, prostate cancer, metastasis, treatment combination

## Abstract

Prostate cancer is the most frequently diagnosed cancer in men and a leading cause of cancer-related death. In recent decades, the development of immunotherapies has resulted in great promise to cure metastatic disease. However, prostate cancer has failed to show any significant response, presumably due to its immunosuppressive microenvironment. There is therefore growing interest in combining immunotherapy with other therapies able to relieve the immunosuppressive microenvironment. Radiation therapy remains the mainstay treatment for prostate cancer patients, is known to exhibit immunomodulatory effects, depending on the dose, and is a potent inducer of immunogenic tumor cell death. Optimal doses of radiotherapy are thus expected to unleash the full potential of immunotherapy, improving primary target destruction with further hope of inducing immune-cell-mediated elimination of metastases at distance from the irradiated site. In this review, we summarize the current knowledge on both the tumor immune microenvironment in prostate cancer and the effects of radiotherapy on it, as well as on the use of immunotherapy. In addition, we discuss the utility to combine immunotherapy and radiotherapy to treat oligometastatic metastatic prostate cancer.

## Introduction

Prostate cancer (PCa) is the most commonly diagnosed cancer in men and the second most common cancer worldwide (1). Despite the fact that more than 70% of cases of localized PCa are cured by local treatments [brachytherapy, (BT), surgery and/or external beam radiation therapy (EBRT)], or are under active surveillance before receiving treatment without altering the benefit of the latter, the median survival of metastatic patients is still less than 5 years (2). Oligometastatic disease (OMD) is first defined by Hellman and Weichselbaum as an intermediate state between local and systemic disease, but there are no validated biomarkers. The ESTRO-ASTRO consensus notes that there are currently no clinical studies showing a lack of benefit beyond a certain number of metastases to define OMD. It is thus a disease where all metastatic sites are treatable by radiotherapy with curative intent. Moreover, OMD can be split in two subtypes: i) synchronous when OMD is detected at the time of the initial diagnosis, ii) metachronous or oligo-recurrent, when OMD is discovered during the course of the disease. Biologically, all metastases are synchronous but our ability to detect them makes them metachronous. Furthermore, metachronous metastases are known to have a better prognosis than synchronous metastases. Elimination of oligometastatic burden by radiation may prevent additional metastatic spread and improve overall survival (3–5). This approach may change the paradigm from palliative to potentially curable disease for oligometastatic PCa patients (6, 7). Indeed, the phase 2 SABR-COMET (Stereotactic ABlative Radiotherapy for the Comprehensive Treatment of OligoMETastases) trial recently evaluated the value of ablative stereotactic radiotherapy to metastatic sites in patients with 1 to 5 metastases (6). There was an overall survival benefit of 13 months (41 *vs* 28) (Hazard Ratio (HR) 0.57, 95% CI 0.3 - 1.1; p= 0.09) in favor of radiotherapy to all sites. Similarly, there was a randomized phase 2 study observation *vs* stereotactic ablative radiation for oligometastatic prostate cancer (ORIOLE) in which 54 patients with recurrent hormone-sensitive oligometastatic PCa were randomly assigned to stereotactic ablative radiotherapy (SABR) or observation in 2:1 ratio (8). At 6 months, disease progression was reported in 19% of patients receiving SABR *versus* 61% of patients undergoing observation (P=.005). The disease progression rate was 11% *vs* 50% (P=.005) and median progression-free survival was not reached *vs* 5.8 months (HR, 0.30; P=.002). Given progress in knowledge and treatments that allow some metastatic patients to be treated with a curative rather than a palliative objective, the concept of oligometastasis is also evolving. The European Society for Radiotherapy and Oncology (ESTRO) and European Organization for Research and Treatment of Cancer (EORTC) consensus seems to relate oligometastases less and less to their number and more to the possibilities of their treatment in terms of technical barriers to the volume and location of metastases (9).

Over the past decade, immunotherapy has revolutionized the treatment of metastatic cancer but has shown only modest efficacy in PCa patients. Nevertheless, recent advances in molecular diagnostics and understanding of immune mechanisms promise to improve the efficacy of immunotherapy in PCa as well. Immunomodulation induced by radiotherapy is a topic of current interest. Indeed, radiotherapy can promote immunogenic cell death and induce the immune response by enhancing antigen cross-presentation and CD8+ cytotoxic T cell response. However, radiation also enhances an immunosuppressive microenvironment by promoting myeloid cells infiltration and macrophage polarization toward an M2-like phenotype, as well as an increase of regulatory T cell subsets involved in the inhibition of naive T cell proliferation and activation (10). Therefore, the combination of radiotherapy and immunotherapy may induce synergistic effects to cure PCa (11). This review aims to highlight the advances in PCa physiopathology and summarize the state-of-the-art knowledge of radiotherapy and immunotherapy in oligo-recurrent PCa.

## The Tumor Immune Microenvironment of PCa

Induction of immune tolerance is a key process throughout tumor development to metastasis. Basically, tumor antigens, neo or not, must be processed and presented by antigen-presenting cells (APCs) such as dendritic cells (DCs). They then migrate to secondary lymphoid organs to activate specific T lymphocyte (T) cells. Conventional CD8α+ DCs appear to be critical APCs for cross-presentation of neoantigens for tumor rejection by T cells (12). Activation of APCs occurs in coordination with other innate immune cells, including natural killer (NK) cells, natural killer T (NKT) cells and γδ T cells in response to damage-associated molecular patterns (DAMPs).

Immunologically, tumors are classified as hot and cold tumors according to their immune infiltrate. Features of hot tumors include increased T cell and cytotoxic T lymphocyte (cTL) infiltration, primarily due to a high tumor mutational burden (TMB), and increased proteins that activate checkpoint proteins. In contrast, features of cold tumors include exhausted cTL cells in the tumor or their absence at the tumor margins, the presence of tumor-associated macrophages (TAM) polarized to an M2-like phenotype (pro-tumor), a low mutational load and poor antigen presentation. PCa can be considered as an immunologically cold tumor (13).

Cancer progression and response to immunotherapy may be strongly influenced by the tumor microenvironment (TME), including immune cells (14). In PCa, tumor-associated antigens (TAAs) are expressed both in normal and tumor cells, but at higher levels in cancer cells. These TAAs are, for example, prostate-specific antigen (PSA), prostate-specific membrane antigen (PSMA), prostatic acid phosphatase (PAP) or CD155. Nevertheless, no anti-tumor response can be triggered due to the immunosuppressive TME (15). Indeed, a lower density of immune cells has been observed in prostatic adenocarcinomas compared to benign nodular hyperplasia of the prostate (16). Anti-tumor CD8+ T cells are also suppressed by the depletion of arginase and tryptophan from the TME after upregulation of secretion of nitrous oxide synthase and indoleamine 2,3-dioxygenase (IDO) by myeloid-derived suppressor cells (MDSCs) (16), or by the presence of a large amount of regulatory T cells (Tregs) compared to other cancers (17), and other immunosuppressive cells such as M2 TAM or neutrophils, both associated with poor survival (18). This immunosuppressive environment is promoted by specific factors such as TGF-β (19) and CXCR2 (20) secreted under the TME. Then, inhibition of CXCR2 may be interesting to improve immunotherapy as tested in a current clinical trial (NCT03473925).

### Focus on the Immune Particularities of the Most Common Metastatic Sites in PCa: Bones and Lymph Nodes

Bones represent 90% of the tumor registry in PCa (21) because they are fertile soil for metastases due to the high blood flow in red bone marrow, interactions between tumor cell and stromal cell, and the production of growth factors, angiogenic factors and bone resorbing factors by stromal cells which allow tumor growth (22). The tumor immune microenvironment is essential for the establishment and growth of PCa bone metastases (23). Disseminated tumor cells secrete IL-6, which attracts TAMs contributing to tumor cell proliferation and angiogenesis in bone sites in an *in vivo* mouse model. A significant concentration of TGF-β is also found in bone metastases that induce the polarization of CD4+ helpers into T reg and may explain the lack of efficiency of immunotherapies in metastatic castration-resistant prostate cancer (mCRPC) (24). Thus, targeting these secreted factors at preferential metastasis sites may be a promising target.

With regard to the lymph nodes, PCa cells build a pre-metastatic niche into them, changing their architecture and immune function (25). In fact, an immunosuppressive microenvironment is established. In PCa patients with pelvic lymph nodes, MDSCs, which include monocytes and granulocytes, exhibit immunosuppressive proteins such as programmed cell death-ligand 1/2 (PD-L1/L2) (26). These MDSCs have an immunosuppression activity and impair the proliferation of CD8+ T cells accumulated in pelvic lymph nodes, which express immune checkpoint proteins. The reactivity of anti-tumor T cells may also be altered since the density of antigens presenting DCs is reduced in the paracortical area (25). Tumor-derived extracellular vesicles (EVs) (discussed in more details in the following section) may be involved in establishing a pre-metastatic niche in lymph nodes by modulating T cells (27). Taking together, the TME cells in PCa metastatic sites favors immune escape and tumor growth (28). The use of immunotherapies to treat prostate metastases is promising to remodel the TME.

## Mechanisms of Immune Escape to Promote Prostate Cancer Development and Metastases

### Prostate Tumor Cells Express Few Tumor Antigens

**Figure 1:**
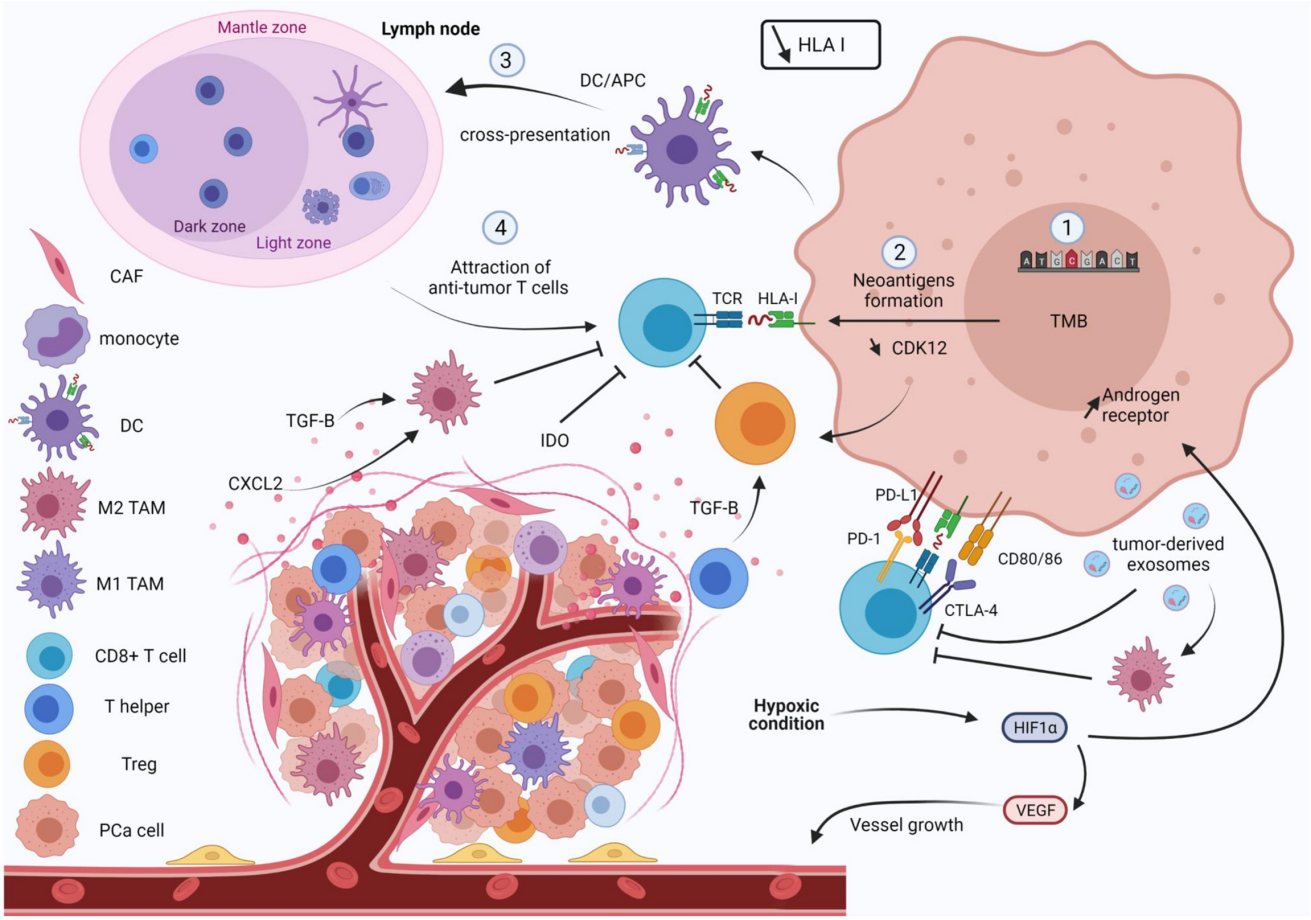
Overview of the physiopathology of prostate cancer. A prostate tumor favors immune escape. Tumor cells harbor a low tumor mutational burden (TMB) and HLA I expression is lost, which decreases the anti-tumor response. Tumor cells also express immune checkpoint inhibitors (PD-L1) such as effector T cells (PD-1 and CTLA-4), leading to exhaustion of cytotoxic T cells. Promotion of immunosuppressive cells such as M2 tumor-associated macrophages or T reg, and suppression of effector T cell activity, are induced by immunosuppressive factors (IDO, CXCL2 and TGFβ). In lymph nodes, expression of PD-L1/L2 by MDSCs establishes a pre-metastatic niche that impairs proliferation of CD8+ T cells. Tumor-derived exosomes are also involved in this immunosuppressive environment by promoting M2 polarization and suppression of CD8+ T cells. Hypoxia molecule HIF-1 is also overexpressed. This induces the expression of the androgen receptor promoting tumor cell growth, notably through remodeling the vasculature. HLA I, human leukocyte antigen; CAF, cancer associated fibroblast; DC, dendritic cell; TAM, tumor-associated macrophage; PCa, prostate cancer.

In cancer cells, various mutations, such as single nucleotide mutations, insertions or deletions, and gene fusions, alter the coding amino acid sequences and could generate new immunogenic antigens called neoantigens, specific for the tumor, so-called tumor-specific antigens (TSAs). Some of these mutant peptides may be presented on the surface of tumor cells and recognized by T cells, which could lead to an immune response. Some cancers are more predisposed to mutation than others and accordingly have a high TMB. PCa is associated with a low TMB (29) and is considered a poorly immunogenic cancer, as this lack of neoantigen formation reduces the ability of TILs (Tumor Infiltrated Lymphocytes) to kill or not to kill tumor cells after cross-priming by APCs (30). Nevertheless, TMB increases with age and tumor characteristics such as a higher Gleason score (31), but also due to the lack of DNA mismatch repair proteins (MSH2/6, MLH1 and PMS2) or proofreading/exonuclease domains such as polymerase epsilon (POLE) or DNA polymerase delta (POLD1) (32). Consequently, prostate tumors with high TMB display a stronger anti-tumor lymphocyte infiltration of memory CD4+ T cells, CD8+ T cells and follicular helper cells (18) (**Figure 1**).

### Loss of HLA I Expression in Prostate Tumor Cells

Loss of HLA (Human Leukocyte Antigen) class I expression is observed in 34% in primary PCa and 80% in lymph node metastases (33). This leads to impaired cTLs response and tumor escape (34). This loss may be due to i) mutation or deletion of structural genes such as heavy chain gene or β2M (beta-2-microglobulin), ii) post-transcriptional and pre-transcriptional regulation of HLA genes especially by non-coding RNA, iii) post-translational mechanisms of HLA protein such as modification of amino acid residues in the peptide-binding groove impairing peptide binding, iv) signaling mechanisms and stimuli from the TME (35) Conversely, radiation therapy could increase HLA class I molecules for many days in a radiation dose-dependent manner (36).

### Prostate Tumor Cells Express Immune Checkpoint Ligands

To escape the anti-tumor immune response, tumor cells increase their expression of immune checkpoint ligands, such as PD-L1. This molecule binds to its receptor, programmed cell death 1 (PD-1), which is expressed by T cells, leading to their anergy. Patients with expression of at least 1% of PD-L1 on tumor cells are associated with shorter metastasis-free survival than those with PD-L1 negative tumors (37). Furthermore, these patients have a fourfold higher risk of developing distant metastases. Another negative regulator of T cells is the cytotoxic T lymphocyte antigen 4 (CTLA-4), which is also upregulated in PCa (38).

### Hypoxia and Epithelial-Mesenchymal Transition

In PCa tumors, pO2 measurements, using an Eppendorf pO2 microelectrodes, showed that increased levels of hypoxia are correlated to clinical stage of the disease (39), and the hypoxic prostate/muscle pO2 ratio predicts biochemical failure in patients (40). The hypoxia-inducible factor 1 (HIF-1), a transcription factor regulated by oxygen, is also overexpressed in PCa and metastases (41). Recurrent PCas are associated with increased expression stability and translocation of the androgen receptor which is also upregulated by hypoxia. Thus, tumor cells are more sensitized to the growth-promoting effect of dihydrotestosterone (DHT) (42). DHT is also implicated in the stabilization of HIF-1a, strengthening the hypoxic response (43). Under hypoxic conditions, HIF-1 induces CD47, overexpressed in many cancers who can bind with SIRPα (signal regulatory protein alpha), an inhibitory receptor which is mostly located on macrophages. The binding of CD47-SIRPα transmits a “don’t eat me” signal, which can prevent cancer cells from immune clearance. Subsequently, expression of CD47 allows tumor cells to increase their stemness and escape phagocytosis. This induces tumor cell progression and increased mortality. Thus, the induction of CD47 in hypoxic tumor cells leads to a disruption of macrophage signaling and does not allow phagocytosis of tumor cells (44). In addition, HIF-1 increases Nanog, which leads *via* TGF beta secretion to an increase in T reg and immunosuppressive macrophages and to a decrease in CD8 T lymphocyte infiltration. Inhibition of Nanog in a hypoxic tumor cell results in a decrease in TGF beta, an increase in CD8 T infiltration and a decrease in immunosuppressive cell infiltration (45).

Hypoxia can induce a certain plasticity in tumor cells, with epithelial cells that can acquire a mesenchymal phenotype, a process called epithelial-mesenchymal transition (EMT). Prostatic adenocarcinomas often show partial cell dissociation with destabilized junctions, corresponding to a grade 3 of 4 of the EMT (46). These grades are defined on three criteria: i) state of cell polarization, ii) stade of cell adhesiveness and iii) expression of intermediate filament proteins. EMT can play a part in immune escape, such as loss of cell-cell recognition, as a decrease in e-cadherin causes modulation of the T cells’ synapse, a structure needed for an efficient immune response, and leads to an overexpression of the PD-L1 increasing immune tolerance (47). Mesenchymal cells also show a decrease of MHC1 expression but they express different factors promoting the differentiation and recruitment of Treg lymphocytes, the differentiation of DCs into immature DCs, and overall lead to immunosuppression in the tumor (47).

## Immunotherapy in Prostate Cancer

**Figure 2:**
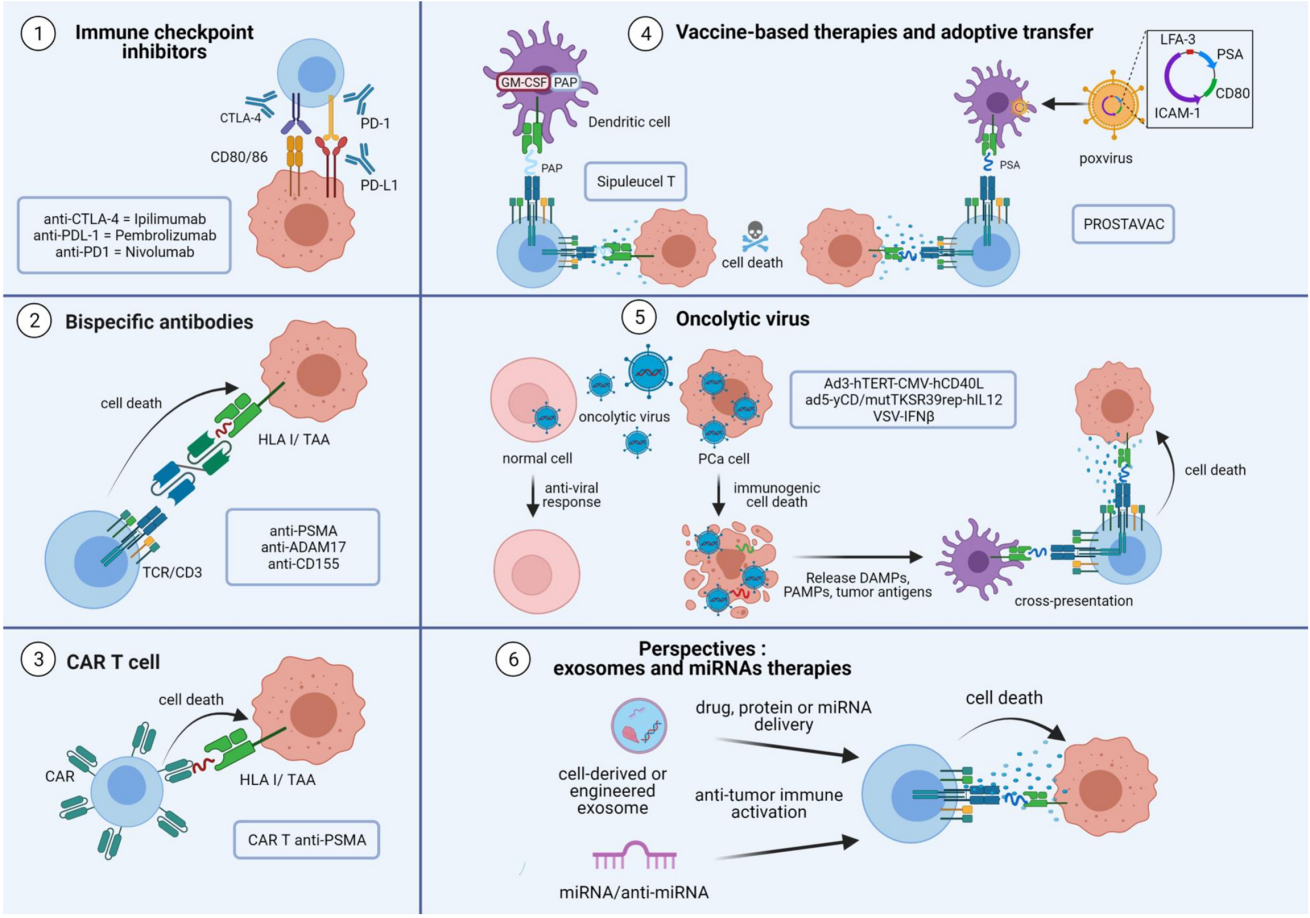
Overview of immunotherapies and perspectives in prostate cancer. The aim of immunotherapies for prostate cancer is to activate tumor-specific CD8+ T cells to induce tumor cell death. The tumor microenvironment induces the expression of immune inhibitory signaling pathways to decrease the cytotoxic CD8+ T cell response. Immune checkpoint inhibitor antibodies directed against PD-L1, PD-1 and CTLA-4 are used to avoid T cell exhaustion. Bispecific antibodies consist of an effective arm that targets the CD3 protein and a target arm that recognizes the tumor antigen. This technology helps CD8+ T cells interact with tumor cells to induce their death. Personalized therapies are also being developed. CD8+ T cells from patients could be manipulated to express a chimeric antigen receptor directed against a specific antigen, notably TAAs. The cells are then expanded and reinjected into the patient, to selectively destroy target cells harboring the surface epitope of interest. Viral and non-viral vectors are used to increase the antigen loading of dendritic cells, leading to an increase in CD8+ cytotoxic T cells in the tumor and response. Enhanced immunotherapy is achieved by the use of oncolytic viruses engineered to replicate only in tumor cells and kill them to induce immunogenic cell death. The use of exosome and microRNA therapies are promising approaches as exosomes and microRNAs are involved in tumor escape. The use of engineered exosomes to deliver proteins, drugs or miRNAs are options to improve anti-tumor response in prostate cancer. TAA, tumor-associated antigen; TCR, T cell receptor; PSMA, prostate specific membrane antigen; CAR, chimeric antigen receptor; PSA, prostate specific antigen; PAP, prostatic acid phosphatase; PCa, prostate cancer; DAMPs, damage-associated molecular patterns; PAMPs, pathogens associated molecular patterns; VSV, vesicular stomatitis virus; MVE, multivesicular endosome.

Immune checkpoint inhibitors (ICIs) are antibodies designed to activate an effective immune response by targeting negative regulators of T cells such as PD-L1, PD-1 or CTLA-4 (48) (**Figure 2**). The use of a CTLA-4-targeted monotherapy, known as ipilimumab, was tested in PCa in an unselected population, but did not result in significant benefit (49). This could be explained by increased expression of PD-1/PD-L1 as a compensatory mechanism that maintains inhibition of the T cell response (50). Alternatively, an anti-PD-1, pembrolizumab, has been commercialized for mCRPC with mismatch repair deficiency and/or microsatellite instability (51), although the relevance of this ICI is still debated. Indeed, the Keynote 19 trial demonstrated that pembrolizumab monotherapy induced antitumor activity in only a small number of mCRPC patients, with an objective response rate (ORR) up to 5% (52). However, in a phase 2 trial (Checkmate 650), double-blockade immunotherapy with nivolumab and ipilimumab showed an ORR of 26% in asymptomatic or minimally symptomatic patients with mCRPC (26). Therefore, determining the subpopulations that might benefit from ICIs’ immunotherapy appears essential.

A promising new approach uses bispecific antibodies (bsAb), also known as bispecific T cell engager (BiTE^®^), to mobilize T cells against tumor cells (53) (**Figure 2**). BsAbs are designed to recognize a TAA with their target arm and to stimulate the T cell receptor (TCR)/CD3 complex with their effector arm (54). Once BsAbs target tumor cells and activate T cells, they induce T cell proliferation and production of cytokines, perforins and granzymes, thereby killing surrounding tumor cells. Various combinations of bispecific conjugates have been tested in PCa. One of the most studied combinations uses PSMA, a specific-prostate antigen whose expression increases with disease progression, and an anti-CD3. Preclinical data demonstrated its efficacy to induce an anti-tumor CD8 T cell response *in vitro, ex vivo* and *in vivo* (55, 56). Several clinical trials with PSMA-targeted T cell engagers (NCT03792841; NCT03577028; NCT03926013) are currently running, particularly for patients with mCRPC (NCT04104607). Furthermore, other TAAs, such as A-disintegrin and metalloprotease 17 (ADAM17) (57) and CD155 (58), are being evaluated as targets of bsAbs in PCa. Interestingly, such therapy may not need to be personalized for each patient.

Chimeric antigen receptor (CAR) T cells are also an interesting tool to fight PCa (**Figure 2**). These therapies are based on re-engineering patients’ T cells to express a TCR directed against a specific tumor antigen. Cells are then expanded and reinjected into the patient to selectively destroy target cells harboring the epitope of interest. Several studies are currently underway with CAR T cells directed specifically against PSMA (NCT04053062; NCT03089203; NCT03873805). Initial results for NCT03089203 demonstrated that adoptive cell transfer of CAR-PSMA-TGFβRdn is safe and feasible in mCRPC patients (59).

Vaccine-based therapies are also being developed to treat PCa (**Figure 2**). In 2010, the Food and Drug Administration approved Sipuleucel-T for the treatment of castration-resistant PCa. Sipuleucel-T is a vaccine based on the transfer of autologous DC to cross-present prostatic acid phosphatase (PAP), a specific prostate antigen, to T cells and active adaptive immune cells (60). Another vaccine therapy evaluated in PCa is PROSTVAC, which uses a genetically engineered poxvirus encoding prostate specific antigen (PSA) to generate a T cell response. It also contained three co-stimulatory molecules: CD80, intercellular adhesion molecule 1 (ICAM-1) and lymphocyte function-associated antigen 3 (LFA-3). However, a phase III study showed no effect on overall survival in mCRPC (61). Combination therapy with a monoclonal antibody directed against PD-L1 and a recombinant vaccine of Avipoxvirus is currently in use (NCT03315871).

A new class of immunotherapy is oncolytic viruses (62, 63) (**Figure 2**). Oncolytic viruses selectively replicate in tumor cells, and induce an immunogenic cell death. The subsequent release of TAAs is thought to trigger an anti-tumor immune response by recruiting DCs and activating T cells. A recent study by Zafar et al. indicated that oncolytic adenoviruses expressing CD40L (Ad3-hTERT-CMV-hCD40L) can effectively stimulate DCs in the immunosuppressive microenvironment of PCa (64). Another genetically engineered oncolytic virus, Ad5-yCD/mutTKSR39rep-hIL12 shows promising effects in preclinical model of PCa through the enhancement of anti-tumor response by cytotoxic immune cells (NK and cTL) (65). In a phase I clinical trial (NCT02555397), improvement in local and metastatic tumor control resulted in significant prolongation of survival.

Extracellular vesicles and microRNA-based therapies represent future perspectives in immunotherapy (66, 67) (**Figure 2**), as they are involved in immunomodulation and tumor progression (68, 69). Exosomes are small extracellular vesicles (50-150nm) formed inside cells (70) and secreted by almost all cell types, including tumor cells. They appear as an interesting tool in cancer immunotherapy due to their low immunogenicity and toxicity (71). However, caution should be taken when targeting exosomes as they are involved in many physiological pathways. They play a role in intercellular communication through a specific interaction between transmembrane proteins of exosomes and receptors on the plasma membrane of recipient cells, and influence physiological and pathological functions in the recipient cell. In PCa, exosomes from cell lines expressing various regulatory proteins such as FAS ligand (FASL) or PD-L1, lead to suppression of T or NK cell responses (19, 72, 73).

Of note, exosomes are naturally enriched with non-coding RNAs such as microRNAs (miRNAs) (74), which are readily transferred to recipient cells (75). miRNAs are a subset of small non-coding RNAs with a length of 19 to 22 nucleotides that regulate gene expression at the post-transcriptional level by translational repression or degradation of the target mRNA. miRNAs included into tumor exosomes (TEX) can participate in tumor immune escape by reducing the CD8+ T cell response (69). Thus, the use of engineered EVs containing miRNA mimic or miRNA antagonists may be a promising therapy to enhance the anti-tumoral response (76–78).

## Radiation Therapy and the Immune Microenvironment of Prostate Cancer

**Figure 3:**
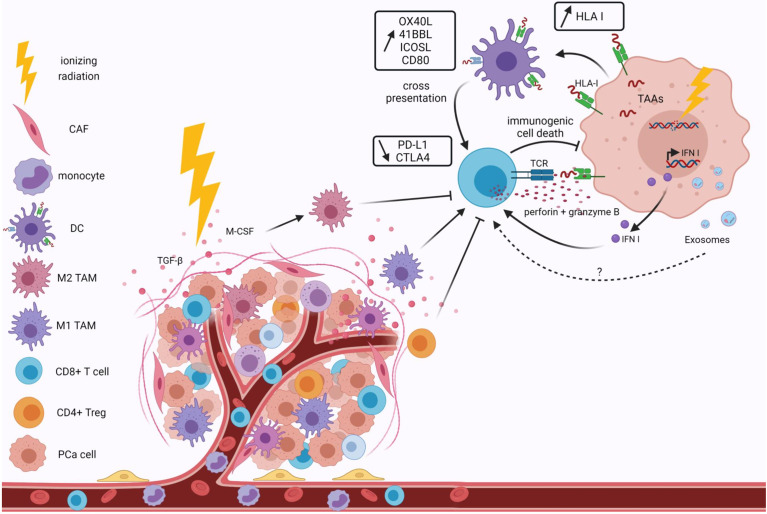
Effects of ionizing radiation on the tumor immune microenvironment. Ionizing radiation modifies the tumor immune microenvironment by recruiting anti-tumor cells. Irradiation remodels the irradiated vasculature to enhance lymphocyte infiltration at the tumor site and macrophage polarization. Irradiation induces DNA damage, leading to the release of tumor-associated antigens, enhanced HLA I expression and type I IFN. An increase in T cell costimulatory molecules and a decrease in inhibitory proteins are also observed after irradiation. This results in immunogenic cell death of tumor cells. Immunosuppressive cells, such as M2 TAM or T reg, are also induced by irradiation due to their more radioresistant phenotypes. They induce suppression of the CD8+ cytotoxic T cell response. All these effects are dependent on the doses and fractionation of irradiation. Extracellular vesicle secretions and contents, notably in miRNAs, are affected by ionizing radiation. Exosomes are involved in tumor immune escape, but the irradiation effects on the promotion of the anti-tumor immune microenvironment of PCa through Evs remain to be addressed. HLA I, human leukocyte antigen; TAA, tumor-associated antigen; IFN I, Type I interferon; TCR, T cell receptor; CAF, cancer associated fibroblast; DC, dendritic cell; TAM, tumor-associated macrophage; PCa, prostate cancer.

Irradiation induces immunogenic cell death leading to the release of tumor antigens, including miRNA patterns (79) or DNA breaks. Radiation therapy also affects the TME, inducing immune cell recruitment and vascular changes (**Figure 3**). Of interest, irradiation of the vasculature may promote infiltration of immune-inflammatory cells [reviewed in (80)]. Conventional 2 Gy dose fractions, a single large dose fraction, or high dose hypofractionated radiotherapy are effective in tumor control. Recent preclinical studies have shown that tumor-resident T cells may be relatively radioresistant and can be amplified to control irradiated tumors (81). The main question remains to determine the dose or fractionation regimen that can transform an immunocompromised tumor into a highly immunogenic one (11). In that regard, stereotactic body radiation therapies (SBRT) can be divided into three categories based on their effects on the immune system or the TME: immunogenic ablative (15-35 Gy fractions), immunomodulatory sub-ablative (8-12Gy), and modulatory low-dose fractions (≈ 2 Gy). Ablative doses lead to profound cell death with concomitant depletion of radioresistant immune suppressor cells in the TME. They may also increase levels of fibrosis and chronic inflammatory/immunosuppressive pathways. However, high ablative fractionation is not considered due to normal tissue tolerance (82). Hypofractionated radiotherapy is considered the most suitable with the goal of immunomodulation, whereas ablative or sub-ablative doses remain more controversial. Preclinical data on fractionation showed that immunomodulatory fractionation of 3 x 8 Gy was more effective than a single ablative dose of 20-30 Gy (83). While modulatory doses (e.g. three x 8 Gy) can produce similar effects to standard fractionation, they resulted in a strong type I interferon (IFN) response (84, 85). On the other hand, low doses of irradiation also have profound effects, leading to remodeling of vessels, reprogramming of macrophages or increased lymphocyte infiltration (86).

Recently, a prospective observational study compared the effects of internal irradiation BT followed by EBRT (15 Gy high-dose rate BT, followed 2 weeks later by 46 Gy in 23 fractions the entire pelvis) with EBRT alone (46 Gy in 23 fractions to the entire pelvis followed by 32 Gy in 16 fractions to the prostate) on immunological cells in PCa patients with Gleason score 9 (87). An enhancement of cTL response was observed in patients receiving BT + EBRT compared to patients receiving EBRT alone, which was associated with IL-2 and granzyme B secretion. In addition, a reduction in CD4+ T cells was observed 3 months after treatment. The authors observed an increase in PD-1 expression by CD4+ and CD8+ cells following radiotherapy. Thus, it might be useful to combine anti-PD-1 checkpoint inhibitors with BT/EBRT to obtain a reliable immunological response.

### Effects of Radiation Therapy on Immunogenic Cell Death and Immune Anti-Tumor Response

Radiation therapy increases DNA damage and leads to activation of IFN I response with pro-inflammatory effects and activation of T cells (11). Radiotherapy also increases antigen presentation (**Figure 3**). Indeed, radiotherapy induces immunogenic cell death, which allows the formation of TAA (88). These TAAs are captured by APCs such as DCs and presented to T cells *via* the major histocompatibility complex (MHC)-I complex, with co-stimulatory signals such as CD80 (89, 90). One of these TAA is the oncofetal tumor antigen 5T4, which is increased by irradiation. This leads to an enhancement of phagocytosis of irradiated tumor cells by DC and thus to an increase in cross-presentation of the 5T4 antigen to CD8+ T cells (91). The number of tumor-specific T cells is increased by radiotherapy in patients (92). In the ORIOLE study, significant clonotypic expansion after SABR was detected by sequencing the T cell receptor (8). A study by Berstein et al. investigated the effects of single dose EBRT on the modulation of costimulatory and co-inhibitory T cell molecules in PCa cell lines (93). The authors observed that irradiation increased the expression of OX40L (OX40 ligand), 4-1BBL (4-1BB ligand) and ICOSL (inducible costimulator-ligand), some of the T cell costimulatory molecules. Furthermore, 72h after irradiation, a decrease in PD-L1 and CTLA-4 expression were observed, as well as an increase in CD8+ T cell activity after their interaction with tumor cells. Thus, irradiation leads to an increase in the expression of co-stimulatory molecules and a decrease of co-inhibitory molecules.

### Irradiation Enhances the Immunosuppressive Environment

After irradiation, an increased amount of immunosuppressive cells (TAM, myeloid derived suppressor cells and Tregs) is also found among the TME, as these cells are more radioresistant than the other immune subtypes (**Figure 3**). A recent study by Lin et al. showed in an allograft PCa model that high-dose radiotherapy induces both immunosuppressive and anti-tumor responses against prostate tumors (94). They observed an increase in MDSCs, followed by an increase in CD8+ TILs. Nevertheless, the response of CD8+ T cells is blocked by Treg. In an *in vivo* model, a systemic increase of MDSCs is observed after irradiation of primary tumor sites (95). The authors showed that the cytokine macrophage colony-stimulating factor 1 (CSF1), also known as M-CSF, increases in irradiated tumors, and in the serum of PCa patients after radiotherapy. This cytokine is involved in M2-like polarization. Therefore, the use of a CFS1 inhibitor in combination with radiotherapy might be interesting. Radiation doses also modulate macrophage phenotypes. Indeed, TAMs can be directed either towards a classical active M1 phenotype by doses below 2 Gy or towards an M2 phenotype with doses higher than 2 Gy. Hypofractionated radiotherapy causes an increase in bone marrow-derived suppressor cells, which are responsible for immune escape from pathogens and tumor malignancy by inducing NK cell and T cell anergy and blocking DC maturation (96). This is problematic because DCs are the main APCs that trigger a T cell response and regulate innate and adaptive immunity. T reg infiltration is also increased in tumors after stereotactic radiotherapy, which correlates with relapse and worsens survival by inhibiting effector T cells, B cells and NK cells (97). Low doses of radiotherapy increase IL-2 and IFN-gamma production, which promotes NK cytotoxicity, while high doses of radiation decrease IL-12 secretion by DCs, which impairs NK cell function. High doses also induce the decrease of Ki67 expression, a proliferative marker, in NK cells within the tumor (96). Finally, tumor-associated neutrophils are certainly a first line of defense against infection and inflammation, but also have pro-tumor effects. Radiation-induced signaling *via* TGF beta leads to the recruitment of these tumor-associated neutrophils, inducing NK anergy.

The interaction between the immune system and the cancer cells is weak and finding the optimal dose and fractionation of radiotherapy to achieve immunogenic results depends on the unique immune properties of each tumor and its TME (11). Thus, the combination of immunotherapy and radiotherapy may be a promising approach to increase the anti-tumor response and avoid immune escape.

## Hormone Therapy and Radiotherapy

In the 1940s, prostate cancers (PCa) were found to have a dependence on androgens. This discovery led to the approach to treat PCa using androgen deprivation therapy (ADT) (98). Moreover, the addition of RT to ADT appears to improve outcomes by enhancing both local and distant disease control (99). Mechanisms of synergy are partially understood, but are likely mediated by the androgen receptor (AR) (100). The AR is a nuclear hormone receptor activated by engagement of its ligands, testosterone and dihydrotestosterone (DHT). Ligand binding exposes the AR in the nucleus, the receptor dimerizes and binds to androgen response elements in the promoter regions of target genes like the PSA (101). Additional co-regulatory proteins are recruited to allow transcription, leading to downstream cellular responses such as growth and survival (102). Thus, androgen ablation therapies repress transcription of AR target genes, which causes activation of tumor cell apoptosis and the eradication of most of the androgen-dependent cancer cells (103) Thus, inhibiting the tumor cell’s ability to repair double-stranded DNA damage by ADT can act as a “radiosensitizer” (104). Combined treatment also induces permanent cell cycle arrest or apoptosis (105). Also, ADT reduces intraprostatic hypoxia which is an important risk factor for poor locoregional disease control and biochemical failure after RT (106, 107)

Finally, enhanced immune responses have also been reported after the association between ADT and RT (108). In addition, change has been also observed in ADT-treated mice CRPC following RT with more TILs associated and an attenuated MDSC recruitment (109). In fact, RT promotes T cell priming by the release of tumor antigens and pro-inflammatory soluble mediators. On the other hand, ADT promotes lymphopoiesis, immune cell trafficking and tumor infiltration (110). Associating immunotherapy to this combination may enhance these processes. Also, there is the question about the precisely timing of immune modulation and depends on many factors, such as the type of ADT, the RT strategy used as a drug (11). In a clinical report, ADT promoted strong adaptive anti-tumor T- and B-cell responses; however, peripheral TH1 and TH17 effector memory subsets decreased after 2 years of treatment (111).

## Hormone Therapy and Immunotherapy in Prostate Cancer

Both preclinical and clinical data showed that androgen-depriving therapy (ADT) synergizes with prostate cancer radiotherapy (100, 112). The impact of testosterone on tumor immune response is ambiguous (113). On the one hand, hormone-naive prostate cancer may respond better to immunotherapy than castration-resistant prostate cancer. In mice, orchiectomy synergizes with immunotherapy, whereas androgen receptor (AR) antagonists suppress the effects of immunotherapy by impairing the adaptive immune responses through interference with initial T cell priming (114). On the other hand, ADT induces T cell infiltration of the prostate (115). Neoadjuvant ADT promotes immune infiltration with proinflammatory effects, but the anti-tumor cells (CD8+ T) are counterbalanced by local pro-tumor cells (TAMs and T reg) (116). ADT also does not increase or diminish PDL1 expression (117). Following ADT and vaccination, prostate cancer-specific T cells expand and develop effector functions (118), suggesting that neoadjuvant ADT may increase the efficacy of immunotherapy. Indeed, the androgen receptor antagonist, enzalutamide, has been tested in phase 2 in combination with immunotherapy and has shown interesting results with a 20% objective response rate (ORR) in patients with mCRPC treated with abiraterone naive chemotherapy (119).

### Association of Immunotherapy and Radiation Therapy in Prostate Cancer

In preclinical data, high dose rate brachytherapy (HDBRT) induced a conversion of 80% of cold prostate tumors into intermediate or warm tumors (120). An increase in survival was also observed in a mouse model of CRPC treated with radiotherapy and either anti PD-1 or anti PD-L1 compared to immunotherapy alone (121, 122) (**Table 1**). In a preclinical model of metastatic PCa, combined irradiation of metastases and anti-CTLA-4 efficiently induced response of T cells and improved both local anti-tumor effects and also distant response, suggesting an abscopal effect (125). Other immunotherapy strategies have shown interesting results when combined with radiotherapy in PCa. A recent preclinical study (*in vitro* and *in vivo* models) used radiotherapy to enhance the activity of a vesicular stomatitis virus (VSV) engineered to express IFNβ (126). IFNβ was expressed by the VSV to reduce viral mediated toxicity to non-transformed cells. Amplification of tumor killing by VSV-IFNβ was observed with the combination of radiotherapy. Also, an increase in adaptive anti-tumor response occurred with the rise in CD8+ T cell numbers.

**Table 1:** Radiotherapy and immunotherapy in mouse models of prostate cancer: Effect on tumor volume and survival.

Authors, years	Cancer model	Therapeutic protocol	Efficacy parameters	Outcomes
Philippou BJ Cancer 2020 (116)	Murine PCa	3 x 5 Gy with or without anti PDL1	Tumor growth delay	No benefit to add anti PDL1 in tumor delay
				Rt increase CD8(+) T-cell, dendritic cell but also TAM and regulatory T-cell genes, upregulate PD-1/PDL1,
Dudzinski J immunother Cancer 2019 (92)	Murine castration resistant PCa	Anti PD1 or Anti PDL1 with or without 20 Gy/2 fractions	Overall survival	Anti-PD-1 or anti-PD-L1 + Rt improved survival
			Abscopal response	Anti PD L1 *vs* Anti PD-L1+RT:
				13 days *vs* 30 days (p=0.0003)
				Anti PD1 *vs* Anti PD1+RT:
				21 days *vs* 36 Days(p=0.0009)
				*Anti CD8 antibody blocked the survival effect*
Hannan Cancer Immunol Immunother 2012 (123)	Murine PCa	RT 10 Gy + *Lm* based PSA vaccine ADXS31-142	Tumor growth delay	Benefit of combination therapy in tumor growth delay (p<0.0001)
Guo Mol Cancer Ther 2012 (124)	Murine PCa	RT 30 Gy/10 Gy fractions during 3 consecutive days + Intratumoral modified dendritic cells (DC)	Tumor growth delay	Benefit of the combination in both tumor growth delay and metastases

Lm, Listeria monocytogenes; PCa, prostate cancer; RT, radiation therapy.

Despite encouraging preclinical experiments, clinical trials in patients combining immunotherapy and radiotherapy failed to improve survival in unselected patients (**Table 2**). Early phase clinical trials showed that the combination of any kind of immunotherapy with radiotherapy to the prostate or to metastases was safe. In some patients, the combination showed encouraging results: increased CD8+ T-cell response to prostate antigens (127, 129), and high complete response rates (131). In this sense a benefit was found in phase I in 3 patients, and good tolerance with HDRBT, androgen-deprivation therapy and nivolumab (127). Local injection of vaccine to the prostate was specifically able to increase PSA-specific T cells (130, 132) but disappointingly did not increase tumor responses in a randomized phase 2 trial (133). Similarly, CTLA-4 blockade using ipilimumab combined with irradiation induced very interesting biochemical responses (134) but failed to improve survival (135). In metastatic castration- and docetaxel-resistant PCa, the CA184-043 phase 3 study comparing ipilimumab *versus* placebo after palliative bone irradiation (8 Gy in 1 fraction) (135) failed to meet its primary endpoint and did not show improvement in overall survival. However, an updated analysis of the study with an additional 2.4 years of follow-up showed that ipilimumab potentially conferred a survival benefit at later stages (136), suggesting that a small subset of patients benefited significantly from ipilimumab. A second study (CA184-095) in patients with mCRPC naïve to chemotherapy showed that ipilimumab was associated with longer median progression-free survival, but unfortunately no survival benefit was shown (49). This negative result may suggest that there is a small subset of patients with mCRPC who are sensitive to ipilimumab, but only after treatment with radiation therapy. Based on preclinical work, these negative results could be interpreted in different ways: irradiation dose too low, too long time between SBRT and ipilimumab, and too few SBRT fractions (137). Interestingly, establishing antitumor immunity against melanoma is enhanced when elimination of regulatory T cells by anti-CTLA-4 antibody precedes radiotherapy (82). It is now recommended that immunomodulatory drugs be started before high dose fractional SBRT for future radioimmunotherapy strategies.

**Table 2:** Clinical studies reporting radiotherapy and immunotherapy in patients with prostate cancer.

Authors, years	Cancer model	Design & pts	Therapeutic protocol	Efficacy parameters	Outcomes
Finkelstein Immunotherapy 2012 (119)	Localized prostate cancer	Non-randomized open label pilot study: 5 pts	EBRT + ADT 28 months +DC injections after fractions 5, 15 and 25	Assessment of immune reaction on biopsy and blood analysis	Increased CD8+ T-cell response
Rodriguez-Ruiz Ann Oncol 2018 (121)	Advanced Cancer	Two cohort pilot study phase I:17 pts, 2 mCRPC	Cyclophosphamide + intradermal monocyte derived dendritic cells (preload with Hiltonol, TNF Alpha and IFN alpha)+ Hiltonol + SABR 24 Gy/3 fractions	Safety	Safe combination; DC local reaction; abscopal effect in one pt
Lilleby, Cancer Immunol Immunother 2017 (122)	Metastatic hormone naive prostate cancer	Dose escalation trial; phase I/IIa: 22 pts, 21 patients received RT	hTERT vaccine UV1 + GM -CSF + EBRT	Safety	Pruritus G1
				PSA	CR 10 pts (45%)
					PSA decline 14 pts (64%)
Slovin Ann Oncol 2013 (127)	mCRPC with disease progression after interruption of ADT having received less than 1 chemotherapy	Non-randomized open label phase I/II: 50 pts	Ipilimumab monotherapy or Ipilimumab + EBRT	PSA evolution	PSA CR: 1 pt
					PSA decline >50%: 8 pts
					Stable disease: 6 pts
Yuan Prostate Cancer Prostatic Dis 2020 (120)	Localized prostate cancer	Open label single group assignment	ADT+ nivolumab and brachytherapy HDR 11.5 Gy x 2 applications + EBRT 40-50Gy 1.8-2Gy fractions	Safety	G3 toxicity: 1 pt
		Phase I/II: 6 pts		PFS	Response: 3 pts
				interval biopsy	Tissue increase in CD8+ and FOXP3+/CD4+ T cells
					increased circulating CD4+ effector T cells in responders
Twardowski Cancer Treat Res commun 2019 (128)	mCRPC	Randomized phase II: 51 pts	Sipuleucel T alone or sipuleucel T after EBRT to metastatic site 30 Gy/3Gy fractions	Systemic immune response	RT did not enhance humoral or cellular response
Gulley Clin Cancer Res 2005 (125)	Localized prostate cancer	Phase II: 30 pts	EBRT 70 Gy with or without vaccine (rV-PSA +rV B7.1) + GM CSF + IL-2	Safety	Safe combination; PSA-specific cellular immune response to vaccine
Lechleider Clin Cancer Res (126)	Localized prostate cancer	Phase II: 36 pts	EBRT + priming dose of vaccinia PSA and vaccinia B7.1 +GM CSF + IL-2 post vaccination	Safety	Safe combination; increase in PSA-specific T cells
Kamrava Prostate Cancer Prostatic Dis 2012 (129)	Localized prostate cancer	Randomized phase II: 36 pts	EBRT + ADT with or without vaccine (two recombinant vectors expressing PSA or human T cell costimulatory molecule B7.1)+Il2	PSA	No difference in PSA control with vaccine *versus* standard treatment
Fizazi, Eur Urol 2020 (130)	mCRPC in progression after docetaxel	Randomized phase III: 799 pts	8 Gy on bone metastases + ipilimumab or placebo	Overall Survival rate	RT + Ipilimumab *versus* Placebo OS rate:
					2 yr: 25.2% *vs* 16.6%
					3 yr: 15.3% *vs* 7.9%
					4 yr: 10.1% *vs* 3.3%
					5 yr: 7.9% *vs* 2.7%

Pts, patients; G, Grade; CR, Complete response; yr, year; mCRPC, metastatic castration-resistant prostate; EBRT, external beam radiation therapy; SABR, stereotactic ablative radiation therapy.

Patient selection might be the key for successful combinations. In patients with early-stage PCa, several studies have evaluated PD-1/PD-L1 as a prognostic marker. High expression of PD-L1 correlated with significantly shorter biochemical recurrence-free survival regardless of tumor stage, PSA, Gleason score and surgical margins (138). Likewise, methylation of PD-1 (123) and PD-L1 (124) promoters has been shown to independently predict biochemical progression-free survival in two independent cohorts. In another cohort of patients receiving salvage radiotherapy after a biochemical relapse, T cells infiltrating the PD-1 expressing tumor predicted relapse (128). A recent study showed that up to 25% of cases of localized PCa express PD-L1, which is correlated with increased density of CD8^+^ T cells and *RB1* and *BRCA2* losses, and deletions of *CHD1* (139), suggesting that a subset of localized PCa is able to stimulate immune responses. **Table 3** summarizes ongoing clinical studies in both localized and metastatic PCa populations. Several studies combine anti PD-1/PD-L1 inhibitors with irradiation, mostly in unselected metastatic patients. Future studies combining immune checkpoint inhibitors and radiotherapy should therefore probably focus on biomarker-selected, especially immune-related and DNA repair gene-deficient, subpopulations of PCa patients.

**Table 3:** Ongoing radiation therapy and immunotherapy in prostate cancer.

Study Number	Study	Number of Patients	Primary outcome
NCT04569461	Trimodality Approach to Unfavorable Localized Prostate Cancer: a Prospective Trial of Neoadjuvant Pembrolizumab, ADT, and Prostate SBRT Followed by Radical Prostatectomy	39	Percentage of subjects who achieve biochemical progression-free survival (BPFS) at 24 months (2 years)
NCT04262154	SAABR: Single Arm Phase II Study of Abiraterone + Atezolizumab + GnRH Analog and Stereotactic Body Radiotherapy (SBRT) to the Prostate in Men with Newly Diagnosed Hormone-sensitive Metastatic Prostate Cancer	44	Failure-free rate at 2 years
NCT03795207	A Randomized Phase II Trial of Stereotactic Body Radiation Therapy (SBRT) With or Without Durvalumab (MEDI4736) in Oligometastatic Recurrent Hormone Sensitive Prostate Cancer Patients	96	Two-year progression-free survival
ACTRN 12619000097145	A phase II, open-label study of durvalumab in combination with stereotactic body radiotherapy in androgen-intact patients with oligometastatic prostate cancer.	30	Freedom from biochemical failure and toxicity
NCT03649841	Radiation Enhancement of Local and Systemic Anti-Prostate Cancer Immune Responses	30	Percent change in peripheral blood effector T-cells (CCR7-/CD45RO)
NCT03543189	Combination of Nivolumab Immunotherapy with Radiation Therapy and Androgen Deprivation Therapy in the Management of Gleason Group 5 Prostate Cancer	34	Phase 1: Safety Run In - Rate of Dose Limiting Toxicity (CTCAE V5.0)/Phase II: Relapse Free Survival Rate
NCT03007732	Phase II Trial Pembrolizumab or Pembrolizumab in Combination with Intratumoral SD-101 Therapy in Patients With Hormone-Naïve Oligometastatic Prostate Cancer Receiving Stereotactic Body Radiation Therapy and Intermittent Androgen Deprivation Therapy	42	Change Rate of prostate-specific antigen (PSA) < nadir + 2 ng/mL from first day of treatment to 15 months (Cohort 2)

ADT, Androgen deprivation therapy; DC, dendritic cells; EBRT, external beam radiotherapy; mCRPC, metastatic castration-resistant prostate cancer.

### Abscopal Effect in Prostate Cancer

The abscopal effect is a rare phenomenon commonly defined by the observation of an objective response at distance from the treated tumor site. Since its initial description in 1953 by Dr. RJ Mole (140) only 46 cases of abscopal responses have been reported until 2016 (141), although many studies have been performed to reproduce this phenomenon with disappointing results. Abscopal response remains one of the most active areas of research in oncology (142).

In a mouse model of castration resistant prostate cancer, an abscopal effect was observed after combining radiotherapy with an anti-PD-1 or an anti-PD-L1 antibodies (121). The authors observed an increase survival and a reduction in tumor graft growth after combining therapies compared with immunotherapy alone. An abscopal response is also observed in clinical trials (143). One patient with mCRPC showed a reduction in non-irradiated metastases after the combination of SABR and DC vaccine. Increased infiltration of CD3+ and CD8+ T cells was also observed.

The combination of radiotherapy with immunotherapy may enhanced frequency of the abscopal response. However, few studies have reported this effect. Therefore, further investigations need to be conducted on the optimal dose/fractionation of RT and the optimal schedule for the administration of RT with immunotherapy elicit the best abscopal response. These studies need to be addressed in future preclinical and clinical trials.

## Conclusion

There is growing evidence that combination of immunotherapy and radiotherapy is a promising strategy to achieve overall survival benefits for patients. Radiation therapy of the primary tumor and/or metastases in combination with immunotherapy increases overall survival in preclinical models of prostate cancer. However, despite clinical evidence of increased immune response, clinical studies have failed to show improved survival following combined immunotherapy and radiotherapy. It appears essential to better understand the mechanisms of metastases and notably the communication between tumor cells and immune cells. These may open up the development of new therapeutic approaches.

## Author Contributions

SS initiated the study. LO and ML performed the scientific literature search and wrote the manuscript. LO designed the tables. ML designed the figures. DF, VP and SS supervised, helped to revise and edit the manuscript. All authors contributed to the article and approved the submitted version.

## Funding

ML was supported by a regional grant (EpiSAVMEN), and by Ms. Suzanne Saulnier. ML and DF were recipients of grant “Ligue contre le Cancer, comités 16, 22, 29, 35, 44, 49 and 56”. ML and DF were also recipients of “Cancéropôle Grand Ouest AO Structurant ExomiR”. SS and VP were recipients of grant “Ligue contre le cancer”, transports Loizeau, De Graet consulting, Astra- Zeneca and Ms. Suzanne Saulnier. This study received funding from Astra Zeneca. The funder was not involved in the study design, collection, analysis, interpretation of data, the writing of this article or the decision to submit it for publication. LO was supported by an institutional grant from CHRU Brest. The POSTCARD F-GETUG P13 study received funding from Astra Zeneca.

## Conflict of Interest

The authors declare that the research was conducted in the absence of any commercial or financial relationships that could be construed as a potential conflict of interest.

## Publisher’s Note

All claims expressed in this article are solely those of the authors and do not necessarily represent those of their affiliated organizations, or those of the publisher, the editors and the reviewers. Any product that may be evaluated in this article, or claim that may be made by its manufacturer, is not guaranteed or endorsed by the publisher.

## Abbreviations

APC, antigen-presenting cell; bsAb, bispecific antibodies; BT, brachytherapy; CAR, chimeric antigen receptor; CSF1, colony-stimulating factor 1; CTLA-4, cytotoxic T lymphocyte antigen; cTL, cytotoxic T lymphocyte; DC, dendritic cell; DHT, dihydrotestosterone; EBRT, external beam radiation therapy; EORTC, European Organization for Research and Treatment of Cancer; EMT, epithelial-mesenchymal transition; ESTRO, European Society for Radiotherapy and Oncology; EV, extracellular vesicle; HIF-1, hypoxia-inducible factor 1; HLA, human leukocyte antigen; HR, hazard ratio; ICI, immune checkpoint inhibitors; IFN, interferon; mCRPC, metastatic castration-resistant prostate cancer; MDSC, myeloid-derived suppressor cell; MHC, major histocompatibility complex; miRNA, microRNA; NK, natural killer; NKT, natural killer T; ORR, objective response rate; PAP, prostatic acid phosphatase; PCa, prostate cancer; PD-1, programmed cell death 1; PD-L1/2, programmed cell death-ligand 1/2; POLD1, DNA polymerase delta; POLE, polymerase epsilon; PSA, prostate-specific antigen; PSMA, prostate-specific membrane antigen; SABR, stereotactic ablative radiotherapy; SBRT, Stereotactic body radiation therapy; T, T lymphocyte; TAA, tumor associated antigen; TAM, tumor associated macrophage; TCR, t cell receptor; TEX, tumor exosome; TIL, tumor infiltrating lymphocyte; TMB, tumor mutational burden; TME, tumor microenvironment; T reg, regulatory T cell; TSA, tumor specific antigen; VSV, vesicular stomatitis virus.
